# The Edge Effect in High-Throughput Proteomics: A Cautionary Tale

**DOI:** 10.1021/jasms.3c00035

**Published:** 2023-05-08

**Authors:** Colleen B. Maxwell, Jatinderpal K. Sandhu, Thong H. Cao, Gerry P. McCann, Leong L. Ng, Donald J.L. Jones

**Affiliations:** † The Leicester van Geest MultiOmics Facility, Hodgkin Building, University of Leicester, Leicester LE1 9HN, United Kingdom; ‡ Department of Cardiovascular Sciences and NIHR Leicester Biomedical Research Centre, Glenfield Hospital, Leicester LE3 9QP, United Kingdom; § Leicester Cancer Research Centre, RKCSB, University of Leicester, Leicester LE2 7LX, United Kingdom

## Abstract

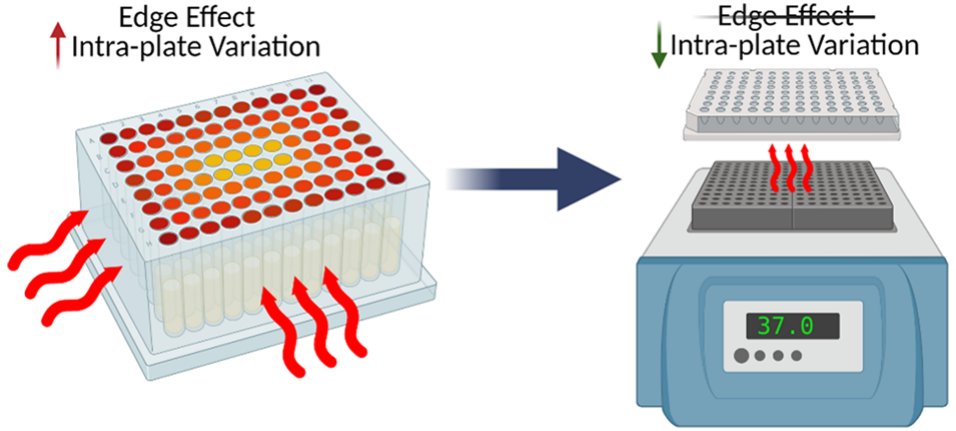

In order for mass spectrometry to continue to grow as a platform for high-throughput clinical and translational research, careful consideration must be given to quality control by ensuring that the assay performs reproducibly and accurately and precisely. In particular, the throughput required for large cohort clinical validation in biomarker discovery and diagnostic screening has driven the growth of multiplexed targeted liquid chromatography coupled to tandem mass spectrometry (LC-MS/MS) assays paired with sample preparation and analysis in multiwell plates. However, large scale MS-based proteomics studies are often plagued by batch effects: sources of technical variation in the data, which can arise from a diverse array of sources such as sample preparation batches, different reagent lots, or indeed MS signal drift. These batch effects can confound the detection of true signal differences, resulting in incorrect conclusions being drawn about significant biological effects or lack thereof. Here, we present an intraplate batch effect termed the edge effect arising from temperature gradients in multiwell plates, commonly reported in preclinical cell culture studies but not yet reported in a clinical proteomics setting. We present methods herein to ameliorate the phenomenon including proper assessment of heating techniques for multiwell plates and incorporation of surrogate standards, which can normalize for intraplate variation.

## Introduction

The emergence of mass spectrometry as a prominent technique for biomarker discovery and validation, diagnostic screening, and other clinical applications is in no small part due to the high-throughput capabilities of liquid chromatography coupled to tandem mass spectrometry (LC-MS/MS). Using LC-MS/MS, hundreds of samples with better statistical power may be analyzed to identify novel biomarker signatures of disease. The transfer of quantitative protein assays to the clinic represents the bottleneck in the biomarker discovery pipeline,^1,2^ with the development of high-sensitivity immunoassays as the current gold standard for clinical protein tests.^3^ However, these have limited multiplexing ability due to cross-reactivity and can also be susceptible to lot-to-lot variation, even for commercial monoclonal antibodies.^3,4^

The large scale required for biomarker validation has thus driven the growth of multiplexed targeted LC-MS/MS assays for clinical applications. Peptide multiple reaction monitoring (MRM) assays typically enable more flexible assay development, better specificity, and greater multiplexing ability than immunoassays.^5^ The high levels of specificity conferred by MRM assays are due to their ability to measure several relevant data points for each peptide: the *m*/*z* of the precursor ion, the *m*/*z* of product ions generated by collision-induced dissociation, and the retention time of the peptide under the specified operating conditions.^6^ The use of multiwell plates coupled with MRM enables high-throughput sample preparation and analysis amenable to automation, which has already been adopted widely by clinical laboratories for the analysis of small molecules, for example in the assessment of vitamin D status^7^ and total testosterone in serum.^8^

Additionally, preclinical multiomics studies coupling genomic data spanning tens of thousands of samples to mass spectrometry measurements require reliable high-throughput LC-MS/MS analysis. In order for LC-MS/MS-based proteomics to continue to grow as a platform of choice for both preclinical studies and the translation of biomarker panels to the clinic, careful consideration must be given to the quality control challenges that arise in such high-throughput analyses. The assay must perform across multiple samples in an accurate and precise way with good preanalytical stability.^9^ Randomization of sample position as well as the use of suitable quality controls across the plate are essential, such as system suitability tests, blanks, positive controls, and negative controls. Even when these quality controls are in place, large scale MS-based proteomics studies may still be plagued by batch effects: artifacts in the data which can introduce noise and confound the detection of true biological differences, resulting in Type I and II statistical errors.^10^ This technical heterogeneity may be due to sample preparation batches, different reagent lots, different analysts or instruments, or indeed MS signal drift.

Many recent publications focusing on large-scale proteomics discovery experiments highlight the value of bioinformatics approaches to assess and process data to combat these often unavoidable batch effects.^11−15^ Much attention has been focused on data dependent (DDA) and data independent (DIA) acquisition discovery experiments, with less focus on the assessment of batch effects on targeted analyses for clinical validation where reliable results free from technical variation are equally critical. Here we sought to investigate technical batch effects within multiwell plates in a high-throughput plasma protein digestion protocol. Interplate variation has previously been characterized in LC-MS/MS and other biomarker discovery platforms such as as Olink (Uppsala, Sweden) and SomaLogic (Colorado, USA);^15−17^ however, intraplate variation is underreported in comparison. Čuklina and colleagues evaluated intraplate variation introduced by a liquid handling system and methods to reduce this effect *in silico*,^11^ but to our knowledge, no previous work has investigated intraplate temperature gradients as a potential source of technical variation in proteomics.

In particular, we report a technical batch effect within multiwell plates termed the “edge effect”, a phenomenon which has not yet been reported in a proteomics study. This effect has been widely reported in cell culture, where increased evaporation occurs from corner and edge wells compared to interior wells due to temperature gradients across the plate.^18−20^ In this work, we present a cautionary tale of the edge effect in high-throughput bottom-up proteomics resulting from thermal gradients which can arise when using multiwell plates, exploring the source of the effect and practical methods to ameliorate it.

## Experimental Section

### Plasma Collection

Plasma was collected from healthy donors with informed consent under Research Ethics Committee (REC) reference: 13/EM/0049. Blood was collected by venepuncture into tubes containing ethylenediaminetetraacetic acid (EDTA) anticoagulant and stored on ice until centrifugation. The blood was centrifuged at 3200 rpm for 20 min at 4 °C using a Sorvall ST 8 Small Benchtop Centrifuge (Thermo Scientific, Loughborough, UK). The centrifuge was allowed to come to a halt on its own and after stopping, the plasma was harvested from the top of the tubes ensuring the pipet tip did not come within 3 mm of the buffy coat layer of white blood cells and platelets. The plasma was stored at −80 °C until analysis. Plasma from two donors was defrosted at room temperature and 5 mL from each donor pooled and aliquoted for digestion.

### Multiwell Plate Plasma Digestion

The plasma digestion protocol was adapted from previous work in the group by Mbasu *et al.* with volumes adjusted for use in multiwell plates.^30^ The general protocol conditions are described herein with adjustments for each experiment outlined in Table 1. In a multiwell plate starting with 10 μL of undepleted human plasma, 75 μL of ammonium bicarbonate (AmBic), 50 mM, pH 7.4, was added. A 5 μL aliquot of 1% RapiGest solution was added, and the plate was sealed and incubated at 80 °C for 1 h in a heater. Reduction was carried out by addition of dithiothreitol to a final concentration of 5 mM followed by incubation for 30 min at 60 °C. The plate was cooled to room temperature (RT), and alkylation was carried out by addition of iodoacetamide to a final concentration of 10 mM. The plate was incubated in the dark at RT for 30 min. Trypsin was added in a 1:25 ratio of trypsin:protein, and the sample was incubated for 18 h at 37 °C. Formic acid (FA) 10% (v/v) was added to a final concentration of 1% (v/v). The plate was centrifuged at 4000*g* for 30 min using an Eppendorf Centrifuge 5810R (Eppendorf, Stevenage, UK), and the supernatant was collected and transferred into Waters QuanRecovery 700 μL Plates (Waters, Milford, USA) for analysis by mass spectrometry.

**Table 1:** Experimental Conditions Employed in “Multiwell Plate Plasma Digestion” Used to Examine the Edge Effect Intraplate Variation in High-Throughput Bottom-Up Proteomics

Experiment	Multiwell Plate	Heater	Plate Lid	Other Changes
**1**	Waters QuanRecovery 700 μL Plates (Waters, Milford, USA)	Incubator Hood TH 30 (Edmund Buhler GmBH, Bodelshausen, Germany)	Corning clear polystyrene 96-well microplate lids (Thermo Fisher Scientific, Loughborough, UK) secured with SLS heat resistant laboratory tape (Scientific Laboratory Supplies, Nottingham, UK)	
**2**	Waters QuanRecovery 700 μL Plates	Incubator Hood TH 30	Waters 96-well 7 mm round plug silicone/PTFE cap mat (Waters, Milford, USA) topped with the Corning lid sealed with tape	
**3**	Waters QuanRecovery 700 μL Plates	Grant SUB6 Universal Water Bath (Grant Instruments, Cambridge, UK)	Waters silicone/PTFE cap mat topped with the Corning lid and sealed with tape	
**4**	Waters QuanRecovery 700 μL Plates	Dry bath heater (Star Lab, Milton Keynes, UK) filled with Bath Armor heating beads (Appleton Woods Ltd., Birmingham, UK)	Waters silicone/PTFE cap mat topped with the Corning lid and sealed with tape	
**5**	Eppendorf twin.tec semiskirted 250 μL 96-well plates (Eppendorf, Hamburg, Germany)	Thermo Scientific Hybaid PX2 thermal cycler (Thermo Fisher Scientific, Loughborough, UK)	Eppendorf Flat Microcap 8-Strips (Eppendorf, Hamburg, Germany)	
**6**	Eppendorf twin.tec semiskirted 250 μL 96-well plates	Thermo Scientific Hybaid PX2 thermal cycler	Eppendorf Flat Microcap 8-Strips	Starting reagents adjusted to 70 μL AmBic and 5 μL bovine serum albumin (BSA, 0.1 mg/mL)

### LC-MS/MS Analysis

LC-MS/MS analysis was performed using a Waters Acquity LC coupled to the Xevo TQ-XS mass spectrometer. The LC was equipped with an Acquity Premier Peptide BEH C18 analytical column, 300 Å, 1.7 μm, 2.1 × 50 mm. Mobile phase A was H_2_O + 0.1% FA. Mobile phase B was acetonitrile (MeCN) + 0.1% FA. The seal wash was H_2_O + 10% methanol (MeOH), the weak needle wash was H_2_O + 0.1% FA, and the strong needle wash was MeCN + 0.1% FA. The flow rate was 0.6 mL/min. The autosampler temperature was 8 °C, and the column temperature was 40 °C. The Xevo TQ-XS was equipped with a Waters Zspray LockSpray in ESI positive mode. The cone voltage was set to 35 V, and the capillary voltage was set to 0.6 kV. The LC gradient program and transitions, collision energies, and scheduling windows for each of the 46 human peptides and BSA peptides measured in the multiplexed MRM assay are available in Supporting Tables S1 and S2. Data was acquired using MassLynx V4.2.

### Data Analysis

Skyline 21.2 Targeted Mass Spectrometry Environment from the MacCoss Lab (Pino *et al*.) was used to generate targeted MRM methods for export to MassLynx 2.4 and import data for analysis.^21^ Production of graphs and statistical analyses were performed using R version 4.2.0 (The R Foundation for Statistical Computing, Vienna, Austria)^22^ on RStudio 2022 (RStudio, Inc., Boston, MA).^23^ Statistical significance of differences was determined using analysis of variance (ANOVA), followed by Tukey’s posthoc test. A *p*-value <0.05 was considered statistically significant.

### Thermal Imaging Analysis

Thermal images were obtained using an FLIR SC600 series infrared (IR) camera (Teledyne FLIR LLC, Kent, UK). Multiwell plates were incubated as described in “Multiwell Plate Plasma Digestion”, removed from the incubator, and immediately photographed to determine the temperature distribution across the plate. Each incubation was performed in triplicate.

## Results and Discussion

### The Edge Effect in Proteomics

**Figure 1:**
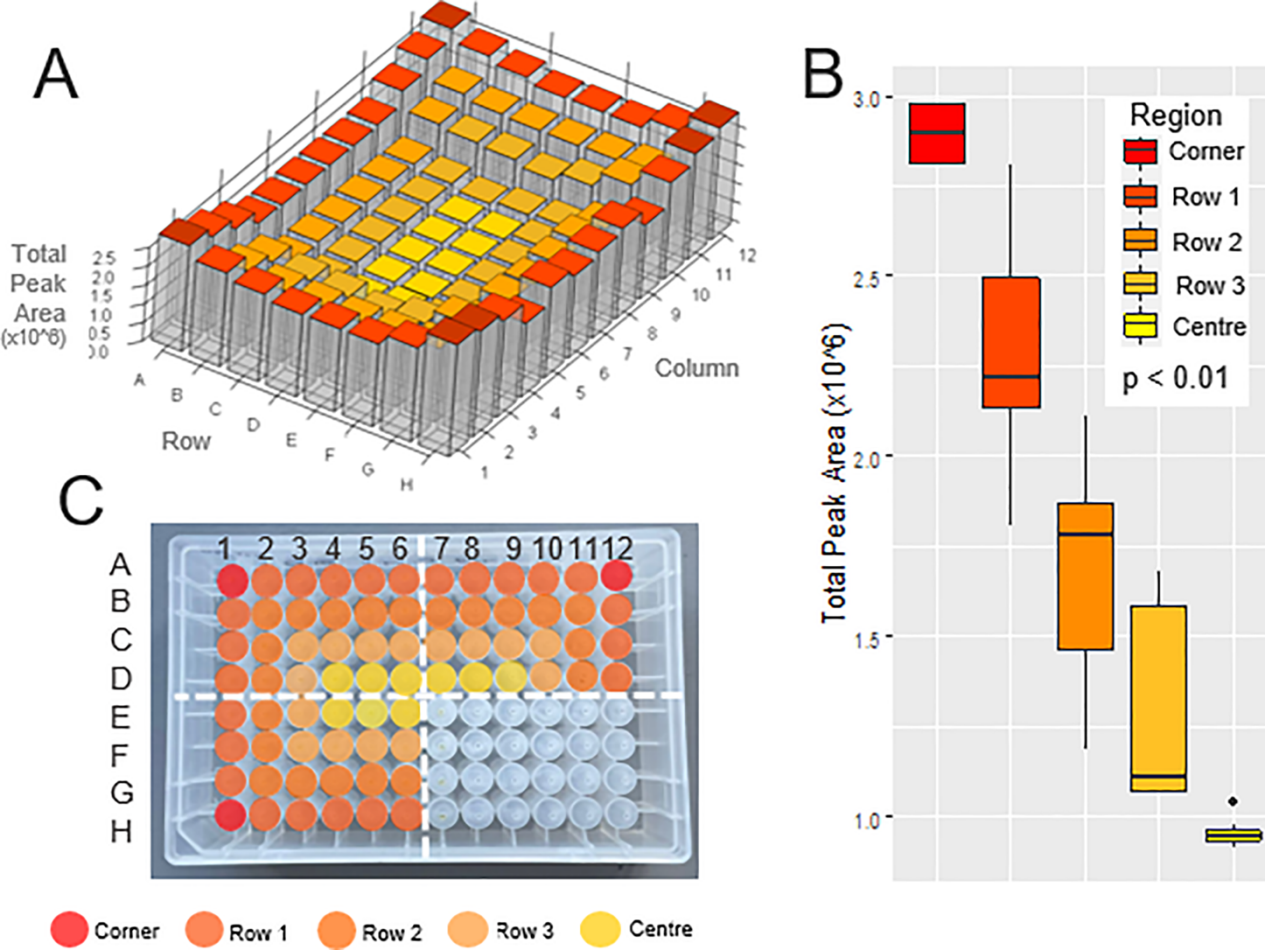
Edge effect in high-throughput proteomics using targeted LC-MS/MS. (**A**) 3D heatmap bar plot illustrating the variation in total peak area across the plate for the peptide NSLFEYQK. A clear edge effect can be seen with significantly higher intensities on the edge wells, particularly the four corner wells, and a gradual decrease in peak intensity into the middle of the plate. (**B**) Boxplots showing comparison in total peak area across five regions of the plate: the corner wells, the rest of the edge wells (row 1), the second row in from the outside (row 2), the third row in (row 3), and the center wells. The peak area between each region is significantly different (*p* < 0.01). (**C**) Assigned regions of the edge effect shown on the deep well plate, with one quadrant blank to show the wells.

**Figure 2:**
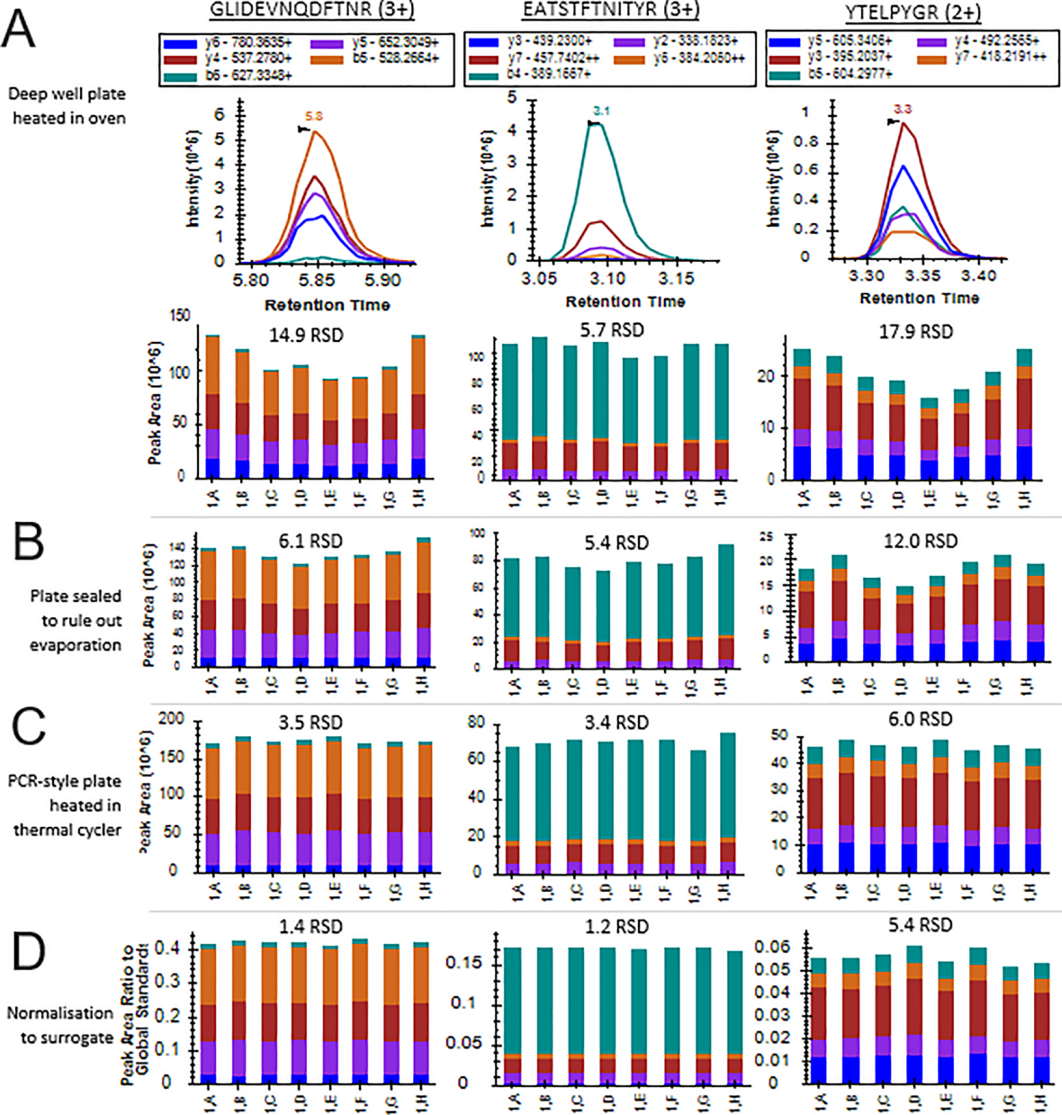
Edge effect and its removal demonstrated in peak area bar plots and RSDs of three peptides (GLI[ . . .], EAT[ . . .], and YTE[ . . .]) across the first column of the plate, i.e., 1,A – 1,H. (**A**) Chromatograms of GLI[ . . .], EAT[ . . .], and YTE[ . . .] and peak areas demonstrating the presence of the edge effect in experiment 1. (**B**) Peak areas for experiment 2, in which the plate was sealed to rule out evaporation from the outermost wells as a cause of the edge effect. (**C**) Peak areas for experiment 5, in which the deepwell plate was replaced with a PCR-style plate heated with a thermal cycler, reducing RSDs compared to the previous experiment and eliminating the edge effect phenomenon. (**D**) Peak areas for experiment 6 utilizing the BSA peptide QTA[ . . .] to normalize for intraplate variation. RSD is reduced for all three peptides compared to the previous experiments.

Intraplate variation was examined in experiment 1 (see Table 1 for conditions) using pooled plasma digested across all 96 wells. Total peak areas of the 46 peptides were measured across the plate using targeted LC-MS/MS. Very high intraplate variation was observed. The average relative standard deviation (RSD) across the 96 wells for all peptides was 38.7%, unacceptably high for biomarker validation and clinical diagnostic screening assays compared to the acceptance criteria recommended by international regulatory guidelines (<15%).^24,25^ The peak areas across the plate are shown in Figure 1A for the most variable peptide NSLFEYQK, which had an RSD of 52.8%. Figure 1A clearly demonstrates higher intensities on the edge wells, particularly the four corners. The total peak area (×10^6^) of the corner wells (mean ± standard error) was 2.9 ± 0.05, the edge wells (row 1) was 2.3 ± 0.05, the second row from the outside (row 2) was 1.7 ± 0.06, row 3 was 1.3 ± 0.06, and the center wells was 1.0 ± 0.01. Comparisons between the regions of the plate revealed a statistically significant difference in peak area (*p* < 0.01) between all regions. The full statistical analysis is shown in Supporting Table S3. This phenomenon of significantly different results in the peripheral wells is termed the “edge effect” and has not yet been reported in a proteomics study. This pattern was conserved across the other peptides, which all had RSDs between 27.5–45.0% across the plate. Figure 2A shows the peak areas and chromatograms annotated with RSDs across the first column of the plate for the three peptides GLI[ . . .], EAT[ . . .], and YTE[ . . .], clearly demonstrating the edge effect pattern. The RSDs of all 46 peptides across the first column of the plate are shown in Supporting Table S4 and Figure S1.

### The Source of the Edge Effect in Proteomics

The proposed mechanism for intraplate variation in cell culture studies is increased evaporation of cell culture medium in the edge wells resulting from regional variation in temperature across the plate.^18−20^ As the medium evaporates, media components and metabolites become differentially concentrated across the plate and alter cell physiology.^19^ A similar effect is observed in ELISA studies where the edge wells may show higher absorbance than the interior wells.^26^ In the conditions of experiment 1, evaporation was visible on the clear polystyrene lid used to seal the plate; therefore, experiment 2 was performed to rule out evaporation due to poorly sealed wells leading to differential concentration of reagents or peptides as a contributing factor to the effect. The plate was securely sealed with a silicone cap mat topped with a clear polystyrene lid and sealed with heat resistant tape. No evaporation was visible on the outer lid following the heating steps, and the volume inside the wells was not found to be different before and after the heating steps.

**Figure 3:**
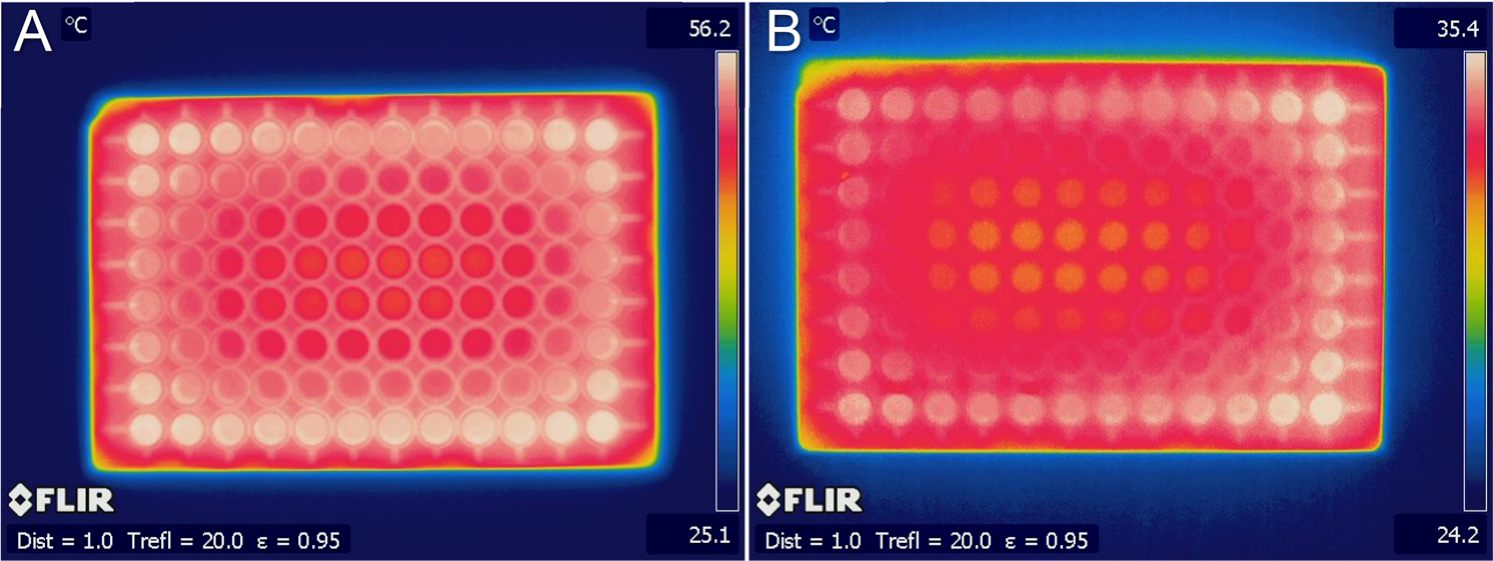
IR images showing the temperature distribution (°C) across the multiwell plates. The temperature is indicated by color, with the scale shown on the index bar on the right-hand side. Temperature is the clear cause of the edge effect in both (**A**) the reduction step and (**B**) the tryptic digestion step. In both experiments, the temperature is highest (white region) in the edge wells of the plate and gradually decreases in temperature into the middle of the plate (red through yellow regions).

In addition, Figure 2B alongside Supporting Table S4 and Supporting Figure S1 demonstrates that the edge effect was still clearly visible in experiment 2; thus, evaporation was ruled out as the cause of the effect in this high-throughput bottom-up proteomics protocol. It was postulated that a thermal gradient across the plate was causing suboptimal temperatures in the inner wells and resulting in less efficient proteolysis. Indeed, Grosch and colleagues have reported the edge effect in the use of multiwell plates for enzymatic assays where thermal gradients in two commercial microtiter plate readers resulted in temperature-induced enzyme activity variation across the plate.^27^ To confirm uneven heating as the cause of the effect and determine whether the reduction or tryptic digestion steps were responsible for the edge effect, IR imaging experiments were performed on the plates after each incubation step. Figure 3A shows the temperatures of the reagents in each of the wells after the reduction step. The maximum temperature reached on the outside of the plate was 56.2 °C, decreasing to approximately 48 °C in the innermost wells of the plate. Figure 3B shows the plate after the tryptic digestion step. The maximum temperature reached on the outside of the plate was 35.4 °C, decreasing to approximately 31 °C in the innermost wells of the plate. In both heated steps, there is a large temperature gradient across the plate which visibly matches the pattern observed in peptide intensities. It can therefore be reasonably concluded that temperature gradients in reduction and tryptic digestion steps are causing differential peptide abundances across the plate and leading to the high RSDs, with less efficient production of tryptic peptides in the inner wells. IR images in Supporting Figure S2 show that the uneven heating pattern is well conserved across triplicate incubations.

### Ameliorating the Phenomenon

Leaving the peripheral row or even the outermost two rows empty to avoid the wells most affected by the phenomenon is standard practice in cell culture;^19^ however, this substantially reduces throughput and capacity and does not address the root cause of the issue. Figure 1B demonstrates heterogeneity between each successive row from the outside of the plate—for high-throughput proteomics in a clinical diagnostic setting, leaving each of these rows empty would be a compromise which negates the use of multiwell plates to achieve increased throughput. Instead, to reduce the thermal gradient across the plate, techniques to directly heat the wells were examined further.

### Minimising Thermal Gradients across the Plate

The use of a water bath in experiment 3 and metallic “bath armor” beads in a dry bath heater in experiment 4 reduced RSD across the wells and improved, but did not eliminate, the edge effect, shown in Supporting Figure S3. Due to the skirting around the plate, it was not possible to seat the plate in the water bath without trapped air accumulating around the perimeter. The heating beads employed in the dry bath in experiment 4 are marketed to replace water in laboratory dry baths and claim to fit to the shape of common laboratory vessels no matter the size or shape, giving greater temperature uniformity than water. However, it was found that the dimensions of the beads resulted in gaps remaining around the perimeter of the plate; much smaller beads would be required to accommodate multiwell plates.

**Figure 4:**
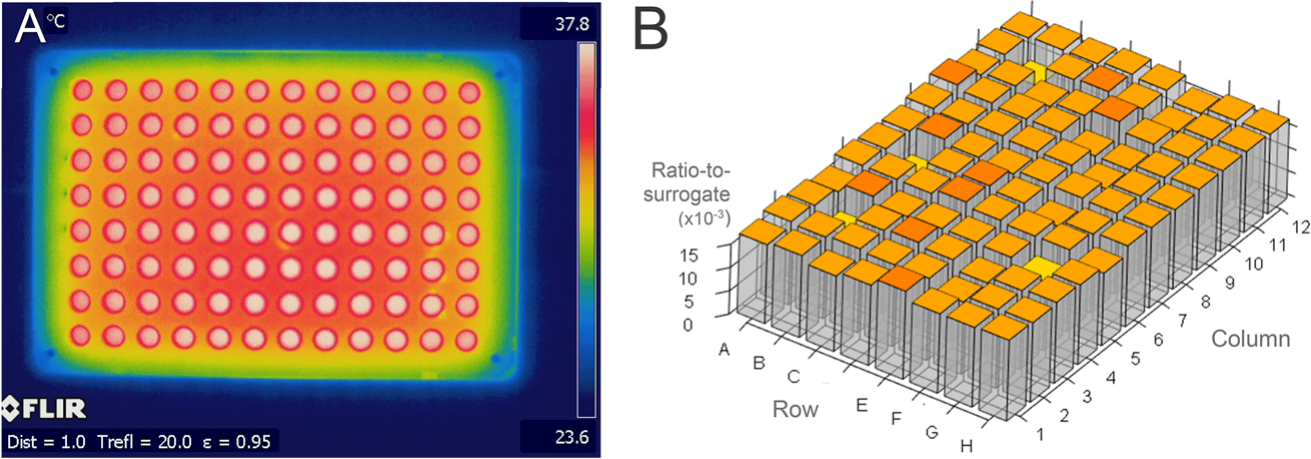
Elimination of the edge effect through use of PCR plates for even heating and normalization to surrogate peptides. (**A**) IR image after the digestion step using a PCR-style plate in a thermal cycler showing a more even temperature distribution across the plate. The temperature is indicated by color, with the scale shown on the index bar on the right-hand side. (**B**) 3D heatmap bar plot illustrating the variation in total peak area across the plate for the most variable peptide NSL[ . . .] in experiment 6. Although a small amount of intraplate variation remains, the edge effect has been eliminated, and RSD across the plate is significantly reduced.

Many vendors have created plates which are marketed to reduce or eliminate the effect in cell culture; however Mansoury *et al.* found that effectiveness in plates from different manufacturers was variable.^20^ Deep well plates are often selected for high-throughput bottom-up proteomics experiments analyzed by LC-MS/MS, since they can hold a relatively large volume (up to 1000 μL) suitable for many experiments and are usually compliant to Society for Laboratory Automation and Screening (SLAS) microplate standards, which means they interface well with liquid handling systems and mass spectrometer autosampler modules. However, the dimensions of the plates can make it difficult for the common laboratory heating equipment previously discussed to make direct contact with the individual vessels for consistent heating across the plate. Thus to ameliorate the edge effect, PCR-style plates heated in a thermal cycler were used to allow direct, even contact with every well (experiment 5). As can be seen in Figure 2C for the peptides GLI[ . . .], YTE[ . . .], and EAT[ . . .], this completely eliminated the edge effect. Reduced RSDs are consistent across all 46 peptides analyzed in the experiment, shown in Supporting Table S4 and Figure S1. To confirm that even heating was resulting in the amelioration of the edge effect, thermal imaging analysis was performed on the PCR plates after the incubation steps, shown in Figure 4A for the tryptic digestion step. There is now little variation in temperature across the plate, and the temperature is also closer to the set temperature of 37 °C compared to Figure 3B, indicating more efficient, accurate heating using PCR-style plates and heaters. Supporting Figure S4 shows a triplicate thermal imaging analysis and even heating in the reduction step.

Rather than the plates themselves resulting in batch effects, it is the ability to apply a thermal gradient robustly and consistently across the whole plate which is of vital importance. Thus, optimum heating conditions for all plates should be assessed during protocol development. Even heating of deep well plates could likely be better achieved using a PCR-style heater with a deep well attachment, which was not available in our facility, such as the C1000 Touch Thermal Cycler with 96–Deep Well Reaction Module (BioRad, Watford, UK) or Eppendorf ThermoMixerC with SmartBlock DWP 500 or 1000 (Eppendorf, Hamburg, Germany).

### Surrogate Standards to Normalize for Intraplate Variation

Although the edge effect pattern was eliminated, a small amount of intraplate variation remained across wells. In targeted LC-MS/MS assays, typically a heavy-labeled synthetic peptide internal standard will be used for quantitation and can correct for sources of variation such as matrix effects, ionization suppression or enhancement, and stability. They cannot, however, correct for intraplate variation arising from sample preparation and digestion inefficiencies. The QconCAT technique uses artificial concatenated peptides, which can be introduced prior to proteolysis and digested with the sample, thus helping to correct for variation arising from sample preparation.^28^ However, QconCATs are relatively expensive and due to a lack of secondary and tertiary structure have been shown to be rapidly and completely digested within a few minutes, much faster than the corresponding proteins.^29^ Thus QconCATs may not be suitable to correct for intraplate variation resulting from variable proteolysis.

We investigated the use of a surrogate standard to normalize for intraplate variation (Experiment 6). BSA was used due to its wide availability and low cost. As an intact protein with higher order structure, it is physiochemically similar to human plasma proteins but is not natively present in the matrix and does not contain structurally homologous peptides to the human peptides being analyzed in this study, thus making it a suitable surrogate standard. An alternative standard should be selected when analyzing potentially homologous peptides from human serum albumin. Normalization to the BSA peptide QTALVELLK was found to be the most globally effective strategy, resulting in an average reduction in RSD of −1.1% across the first column of the plate. The effect of normalization on GLI[ . . .], EAT[ . . .], and YTE[ . . .] is shown in Figure 2D and for the other 46 peptides analyzed in the experiment in Supporting Table S4 and Figure S1.

The average RSD across the 96 wells for all peptides using surrogate peptides (experiment 6) was 4.2 compared to 38.7 in experiment 1, an almost 10-fold reduction. The peak areas across the plate are shown in Figure 4B for the most variable peptide NSL[ . . .], which had an RSD in experiment 1 of 52.8%, reduced to 5.7% in experiment 6. The total peak area normalized to BSA (×10^–3^) of the corner wells (mean ± standard error) was 17.0 ± 0.1, the edge wells was 16.8 ± 0.1, the second row from the outside was 17.0 ± 0.2, row 3 was 16.9 ± 0.2, and the center wells was 17.5 ± 0.3. Comparisons between all regions of the plate revealed no statistically significant difference in peak area (*p* > 0.01). The full statistical analysis is shown in Supporting Table S5. The small amount of stochastic variation across the wells which remains is likely due to other sources of technical variation, for example pipetting error.

## Conclusion

There is an opportunity for mass spectrometry to be a dominant analytical technique in translational and clinical arenas. It is a high-throughput, multiplexable technique that has been demonstrated to be highly reproducible over many hundreds of injections. However, in order for MS to continue to grow as the technique of choice in the clinical laboratory, it is important that every aspect of the workflow—from sample preparation to analysis—is adequately assessed for issues which may compromise the quality and reproducibility of data. In recent years, much attention has been given to assessing and removing sources of technical variation in high-throughput MS studies, since these can introduce noise and confound the detection of true biological differences. Here we presented a cautionary tale of the intraplate edge effect in high-throughput bottom-up proteomics utilizing microplates. We recommend that particular attention is paid to quality control of batch effects not just in DIA and DDA bottom-up proteomics data but also in targeted LC-MS/MS analyses in multiwell plates moving toward formats which can be feasibly adopted by clinical laboratories in large cohort validations or diagnostic screening tests.

We propose that, in addition to other study design factors such as sample randomization across plates, laboratories give careful consideration to conditions for heating multiwell plates (and indeed racks, holders, or other vessels). It is the application of heat robustly and consistently across the whole plate which is of importance, rather than the plates themselves leading to imprecision. Thus, the potential presence of temperature gradients should be carefully assessed for all labware, particularly if the vessels which are used cannot easily be directly heated individually. Otherwise, technical heterogeneity may arise in the data. This applies not only to those performing proteomics studies but across the spectrum of clinical mass spectrometry disciplines which may use multiwell plates to achieve high-throughput. We present methods herein for protocol optimization if temperature gradients are found, including switching to PCR-style heaters where individual wells can be easily directly heated and incorporating surrogate standards which can normalize for intraplate variation.

## Data Availability

Data and transition lists are deposited at The PeptideAtlas SRM Experiment Library (PASSEL) unique identifier PASS04819.

## Supporting Information Available

LC gradient program; SRM transition lists; full statistical analysis results (ANOVA); supporting figures for water bath and bath bead heating experiments; supporting figures for additional replicates of thermal imaging experiments (PDF)
