# Supplementary material for: The Edge Effect in High-Throughput Proteomics: A Cautionary Tale
Source: J Am Soc Mass Spectrom. 2023 May 8;34(6):1065–72. doi: 10.1021/jasms.3c00035 (PMC10251511; doi:10.1021/jasms.3c00035)
Supplement: Supplementary file 1 — js3c00035_si_001.pdf [file js3c00035_si_001.pdf]

SUPPORTING INFORMATION

The Edge Effect in High Throughput Proteomics: A Cautionary Tale

Colleen B. Maxwell[1,2]*, Jatinderpal K. Sandhu[1,2], Thong H. Cao[1,2], Gerry P. McCann[2], Leong L. Ng[1,2], Donald J.L. Jones[1,2,3]

(1) *The Leicester van Geest MultiOmics Facility*, Hodgkin Building, University of Leicester, Leicester, LE1 9HN, United Kingdom.
(2) *Department of Cardiovascular Sciences and NIHR Leicester Biomedical Research Centre*, Glenfield Hospital, University of Leicester, Leicester, LE3 9QP, United Kingdom.
(3) *Leicester Cancer Research Centre*, RKCSB, University of Leicester, Leicester, LE2 7LX, United Kingdom.

*Corresponding author. Email: cbm11@leicester.ac.uk.

Table S1. 12.5 min gradient programme used on the Waters Acquity ultra performance liquid chromatography (UPLC) system for LC-MS/MS data acquisition.

Time (min)	% A	% B
**0.0**	95.0	5.0
**1.5**	95.0	5.0
**8.5**	70.0	30.0
**11.0**	5.0	95.0
**11.5**	5.0	95.0
**11.5**	95.0	5.0
**12.5**	95.0	5.0

Table S2. SRM assay parameters (transitions, CE, scheduled RT windows) for the 46 peptides and BSA peptides.

Peptide	Species	Precursor *m/z*	Frag-ment	Fragment *m/z*	CE (eV)	RT Window (mins)	RT (mins)
**QGFILSGVK (+2)**	*Homo sapiens*	474.8	b4	446.2	21	4.4 - 5.2	4.8
			y5	503.3	21		
			y6	616.4	19		
			y7	763.5	15		
			b8	802.4	11		
**GAFQQAA QILLHAR (+3)**	*Homo sapiens*	508.6	y4	496.3	21	5.0 - 5.8	5.4
			y9	496.8	21		
			y10	560.8	17		
			y11	624.9	19		
			y12	698.4	23		
**DVEAPQIN C(cam)PK (+3)**	*Homo sapiens*	424.2	b7	377.2	22	2.4 - 3.2	2.8
			y3	404.2	16		
			b4	415.2	10		
			y7	428.7	16		
			b5	512.2	18		
**C(cam)ALLL QEIPAISYR (+2)**	*Homo sapiens*	824.0	y8	948.5	25	7.6 - 8.4	8.1
			y9	1076.6	23		
			y10	1189.7	21		
			y11	1302.7	27		
			y12	1415.8	25		
**GLIDEVNQ DFTNR(+2)**	*Homo sapiens*	760.9	y5	652.3	23	5.2 – 6.0	5.6
			y6	780.4	23		
			y7	894.4	25		
			y8	993.5	25		
			y10	1237.5	25		
**DSHSLTTNI MEILR(+3)**	*Homo sapiens*	543.9	y3	401.3	16	7.5 - 8.3	7.9
			y4	530.3	16		
			y5	661.4	14		
			y6	774.5	16		
			b8	856.4	14		

**STVLTIPEIII K(+2)**	*Homo sapiens*	663.9	b5	502.3	23	6.5 - 7.3	6.9
			y11	620.4	25		
			b7	712.4	27		
			y6	712.5	27		
			y9	1039.7	17		
**QIGGDGM MDITDTYK (+3)**	*Homo sapiens*	548.9	b9	453.2	16	5.2 – 6.0	5.6
			y8	493.7	16		
			b10	509.7	16		
			b6	528.2	12		
			y6	740.4	14		
**TDNDGLGF R(+2)**	*Homo sapiens*	497.7	y3	379.2	23	3.2 – 4.0	3.6
			b4	446.2	23		
			y8	447.2	19		
			y4	492.3	13		
**SGIAYILHLK**	*Homo sapiens*	372.2	y5	312.2	18	4.0 - 4.8	4.4
			y6	393.7	16		
			y3	397.3	18		
			y7	429.3	8		
			y7	857.5	10		
**GALHDENT C(cam)NR (+3)**	*Homo sapiens*	429.5	b4	379.2	16	1.4 - 2.2	1.8
			y7	454.7	12		
			b9	499.7	16		
			b7	737.3	16		
**EATSTFTNI TYR(+3)**	*Homo sapiens*	468.6	y5	333.7	23	4.3 - 5.1	4.7
			y2	338.2	21		
			y3	439.2	23		
			b5	490.2	25		
**IIEVEEEQE DPYLNDR (+3)**	*Homo sapiens*	664.3	b4	455.3	17	4.0 - 4.4	4.4
			y10	639.8	23		
			y13	818.4	17		
			y7	892.4	27		
			b15	908.9	17		
**C(cam)VNS PGSFR(+3)**	*Homo sapiens*	341.8	y2	322.2	7	1.2 – 2.0	1.6
			b4	374.1	15		
			y10	432.2	13		
			y13	461.2	11		
			y7	650.3	17		
**GVTSVSQIF HSPDLAIR (+3)**	*Homo sapiens*	609.7	y4	472.3	27	6.5 - 7.3	6.9
			y6	684.4	23		
			y12	692.4	15		
			y13	741.9	19		
			y14	785.4	17		
**FQPTLLTLP R(+2)**	*Homo sapiens*	593.4	y3	385.3	27	6.3 - 7.1	6.7
			y4	486.3	17		
			y5	599.4	25		
			y6	712.5	25		
			y8	910.6	17		

**ALTGHLEEV VLALLK(+3)**	*Homo sapiens*	536.0	y4	444.3	22	7.1 - 7.9	7.5
			y11	632.4	14		
			y12	660.9	14		
			y14	768	22		
**GVDEATIID ILTK(+3)**	*Homo sapiens*	463.3	y8	471.8	23	7.9 - 8.7	8.3
			b5	472.2	23		
			b6	573.3	13		
			y5	589.4	11		
			y6	702.4	11		
**LQETSNWL LSQQQAD GSFQDPC (cam)PVLD R(+3)**	*Homo sapiens*	1044.8	y5	300.2	36	8.0 - 8.8	8.4
			y7	428.7	34		
			y18	1024.5	30		
			y20	1137.5	34		
			b20	1139	36		
**VVEEQESR VHYTVC(ca m)IWRNGK (+3)**	*Homo sapiens*	830.4	b12	729.4	35	5.5 - 6.3	5.9
			y12	766.9	25		
			y14	888.5	27		
			y16	1017	25		
			y8	1032.5	33		
**ILESELEEQL SQHR(+3)**	*Homo sapiens*	571.0	y4	527.3	17	4.2 - 5	4.6
			y9	570.3	11		
			y11	678.3	17		
			y12	742.9	23		
			y13	799.4	15		
**EEFQQEIQ R(+2)**	*Homo sapiens*	603.8	y2	303.2	29	3.7 - 4.5	4.1
			y7	474.7	27		
			y4	545.3	27		
			y5	673.4	25		
			y6	801.4	23		
**DQVLVSC (cam)DTGY K(+2)**	*Homo sapiens*	692.8	y4	468.2	29	3.6 - 4.4	4.0
			y11	635.3	27		
			b6	642.3	25		
			y6	743.3	21		
			y7	830.3	27		
**APGELEHG LITFSTR(+3)**	*Homo sapiens*	543.3	y5	611.3	22	3.9 - 4.7	4.3
			y6	724.4	14		
			y13	730.4	14		
			y14	778.9	22		
			b9	904.5	22		
**SLGTIQQC (cam)C(ca m)DAIDHC(cam)R(+3)**	*Homo sapiens*	683.0	y11	724.3	25	7.4 - 8.2	7.8
			y12	788.3	23		
			y13	844.9	21		
			y15	923.9	27		
			y10	1319.5	19		
**DQNILLGTT YR(+3)**	*Homo sapiens*	431.9	y3	439.2	20	4.3 - 5.1	4.7
			b4	471.2	18		
			y4	540.3	20		
			b5	584.3	20		
			y5	597.3	18		

**GFYFSRPAS R(+2)**	*Homo sapiens*	594.3	b9	507.2	25	3.7 - 4.5	4.1
			b4	515.2	25		
			y5	586.3	21		
			b8	926.5	27		
**FFQYDTWK (+2)**	*Homo sapiens*	567.8	b3	423.2	22	6.2 - 7	6.6
			y7	494.2	18		
			b7	712.3	24		
			y6	840.4	24		
			y7	987.5	22		
**IIMYDQNH LLGR(+2)**	*Homo sapiens*	736.9	y9	558.3	24	7.1 - 7.9	7.5
			y10	623.8	30		
			y11	680.3	30		
			y9	1115.6	30		
			y10	1246.6	24		
**GNFYEASD WFK(+2)**	*Homo sapiens*	682.0	y3	464.3	29	7.1 - 7.9	7.5
			y4	678.3	29		
			b7	836.5	17		
			y7	895.3	19		
			b9	1020.6	29		
**LTQLNLDR (+3)**	*Homo sapiens*	324.9	b3	343.2	14	2.9 - 3.7	3.3
			y7	430.2	10		
			b4	456.3	18		
			b5	570.3	16		
**GQDLLSTV SIR(+2)**	*Homo sapiens*	594.8	b4	414.2	25	3.6 - 4.4	4.0
			b5	527.3	21		
			b8	814.4	27		
			b9	901.5	21		
**HDFFLQGF TPDR(+3)**	*Homo sapiens*	493.9	y3	387.2	10	6.9 - 7.7	7.3
			y4	488.2	14		
			b4	547.2	14		
			b5	660.3	14		
			y6	692.3	14		
**LGNFFINEA MELK(+3)**	*Homo sapiens*	509.3	y3	389.2	17	9.3 - 10.1	9.7
			y7	417.7	21		
			b4	432.2	23		
			b6	692.4	15		
**LQGLEQEA ENK(+3)**	*Homo sapiens*	629.8	y6	355.2	22	2.2 - 3	2.6
			y4	422.2	23		
			y8	480.7	21		
			b9	515.2	25		
			b5	568.2	22		
**HVQGPEPA K(+2)**	*Homo sapiens*	481.8	y3	315.2	19	1.2 - 2	1.6
			y6	598.3	25		
			b6	648.3	21		
			y7	726.4	25		
			y8	825.4	11		
**DLDFDWH K(+3)**	*Homo sapiens*	359.2	b5	303.6	12	3.4 - 4.2	3.8
			b3	344.1	12		
			b6	396.7	14		
			y6	424.2	10		
			b7	465.2	10		

**ALYNQYLQFK(+3)**	*Homo sapiens*	429.9	y3	422.2	16	4.9 - 5.7	5.3
			y4	535.3	18		
			y8	552.3	18		
			b5	590.3	12		
			y5	698.4	14		
**LIINSLYK (+2)**	*Homo sapiens*	482.3	y2	310.2	23	5 - 5.8	5.4
			b3	340.3	23		
			b7	409.2	21		
			y7	425.8	21		
			b4	454.3	23		
**LISLTDENALSGNEELTVK(+3)**	*Homo sapiens*	682.7	y12	637.8	19	7.2 - 8	7.6
			y13	702.4	27		
			b17	900.9	19		
			y9	976.5	21		
			b10	1070.6	27		
**NPC(cam)QDPYILTPENR(+3)**	*Homo sapiens*	572.9	y4	515.3	21	4.3 - 5.1	4.7
			b5	615.2	27		
			y5	616.3	27		
			y6	729.4	15		
			b8	988.4	27		
**GSFAC(cam)QC(cam)PPGYQK(+2)**	*Homo sapiens*	750.3	y6	689.4	33	0.9 - 1.7	1.3
			b7	811.3	31		
			y7	849.4	23		
			y8	977.5	31		
			y9	1137.5	27		
**YTELPYGR (+2)**	*Homo sapiens*	499.8	y3	395.2	21	2.7 - 3.5	3.1
			y7	418.2	17		
			y4	492.3	13		
			b5	604.3	19		
			y5	605.3	19		
**LVSLSAQNLVDC(cam)STEK(+3)**	*Homo sapiens*	588.6	y9	355.8	30	3.4 - 4.2	3.8
			y6	370.2	20		
			y8	476.2	20		
			b5	500.3	24		
			y12	676.3	18		
**LHISPSNMTNQNTNEYLEK(+3)**	*Homo sapiens*	745	y3	389.2	31	5.2 – 6.0	5.6
			b4	451.3	25		
			y4	552.3	27		
			b6	635.4	27		
			b9	981.5	27		
**DGIDDESYEAIFKPVMSK(+3)**	*Homo sapiens*	682	y4	464.3	29	7.1 - 7.9	7.5
			b12	678.3	29		
			y7	836.5	17		
			b8	895.3	19		
			y9	1020.6	29		
**LVNELTEFAK(+3)**	*Bos taurus*	388.5	y3	365.2	11	1.6 - 2.4	2.0
			b7	400.2	17		
			y7	419.2	13		
			b8	473.7	11		
			b9	509.3	11		

**HLVDEPQN LIK(+3)**	*Bos taurus*	435.9	b6	346.2	10	1.4 - 2.2	1.8
			y6	356.7	16		
			y3	373.3	18		
			b10	580.3	14		
			b5	594.3	12		
**YLYEIAR (+2)**	*Bos taurus*	464.3	y3	359.2	20	3.4 - 4.2	3.8
			b3	440.2	10		
			y4	488.3	14		
			y5	651.3	12		
			y6	764.4	16		
**TVMENFVA FVDK(+3)**	*Bos taurus*	467.2	y3	361.2	21	2.7 - 3.5	3.1
			b7	411.2	13		
			y7	413.2	11		
			b8	446.7	13		
			b7	821.4	17		
**LGEYGFQN ALIVR(+3)**	*Bos taurus*	493.9	b10	463.2	18	1.3 - 2.1	1.7
			b9	490.7	22		
			b10	547.3	20		
			y10	590.8	20		
			y6	685.4	20		
**QTALVELLK (+2)**	*Bos taurus*	507.8	y3	373.3	12	5 - 5.8	5.4
			b4	414.2	12		
			b5	513.3	20		
			b6	642.3	18		
			b7	755.4	14		
**YIC(cam)D NQDTISSK (+2)**	*Bos taurus*	722.3	y11	640.8	20	1.4 - 2.2	1.8
			b6	794.3	30		
			y9	1007.5	22		
			b8	1010.4	20		
			y10	1167.5	22		

Table S3. ANOVA and Tukey post-hoc test results comparing the peak areas between each region of the plate: the corner wells, other edge wells (row 1), 2nd from the outside row (row 2), row 3, and centre wells. Significance codes: 0 '***' 0.001 '**' 0.01 '*' 0.05 '.' 0.1 'N/A' 1.

Comparison	p-value (adjusted)	Significance
**ANOVA All Areas ~ Region**	< 0.001	***
**Corner – Centre**	< 0.001	***
**Row 1 – Centre**	< 0.001	***
**Row 2 - Centre**	< 0.001	***
**Row3 - Centre**	0.008	**
**Row 1 – Corner**	< 0.001	***
**Row 2 – Corner**	< 0.001	***
**Row 3 – Corner**	< 0.001	***
**Row 2 – Row 1**	< 0.001	***
**Row 3 – Row 1**	< 0.001	***
**Row 3 – Row 2**	< 0.001	***

Table S4. RSDs of each of the peptides analysed in the experiments shown in Figure 2. The average RSD across all peptides for each experiment is also shown.

Peptide	RSD in each Experiment			
	A) Deep well plate heated in oven	B) Plate sealed to rule out evaporation	C) PCR-Style plate heated in thermal cycler	D) Normalisation to Surrogate
Average (all peptides)	12.4	10.1	4.8	3.7
ALTGHLEEV VLALLK	14.9	15.3	4.3	4.3
ALYNQYLQ FK	13.3	7.2	6.0	3.7
APGELEHGL ITFSTR	20.4	10	4.7	3.7
C(cam)ALLL QEIPAISYR	18.8	10	3.7	3.2
C(cam)VNS PGSFR	9.0	5.2	2.7	2.5
DGIDDESYE AIFKPVMSK	9.1	5.3	4.6	6.5
DLDFDWHK	12.2	2.1	2.8	2.7
DQNILLGTT YR	10.2	3.6	1.7	3
DQVLVSC(c am)DTGYK	10.3	7.5	7.6	2.9
DSHSLTTNI MEILR	9.2	4.5	4.1	4

DVEAPQINC(cam)PK	10.9	6.6	8.9	7.9
EATSTFTNITYR	5.7	5.4	3.4	1.2
EEFQQEIQR	8.6	21.8	7.1	4.4
FFQYDTWK	19.5	16.4	5.4	4.3
FQPTLLTLPR	12.5	24.4	5.4	4
GAFQQAAQILLHAR	5.7	8.1	4.7	2.3
GALHDENTC(cam)NR	12.3	15.3	7.7	4.9
GFYFSRPASR	10.8	5.7	3.1	5
GLIDEVNQDFTNR	14.9	6.1	3.5	1.4
GNFYEASDWFK	9.6	15.9	3.6	2.7
GQDLLSTVSIR	16.6	6.0	5.5	6.9
GSFAC(cam)QC(cam)PPGYQK	5.1	7.7	3.3	2.2
GVDEATIIDILTK	15.7	6.3	8.3	4.5
GVTSVSQIFHSPDLAIR	13	5.4	4.9	2.4
HDFFLQGFTPDR	10.8	14.2	4.5	2.9
HVQGPEPAK	17.2	14.5	4.5	2.9
IIEVEEEQEDPYLNDR	17	8.2	3.4	2.9
IIMYDQNHLLGR	27.1	4.8	6.2	2.7
ILESELEEQLSQHR	17.5	15.3	2.9	2.3
LGNFFINEAMELK	11.1	3.4	2.9	1.6
LHISPSNMTNQNTNEYLEK	12	11.3	5.8	3.2
LIINSLYK	11.5	20.6	7.6	3.4
LISLTDENALSGNEELTVK	11.9	4.3	2.7	7.1
LQETSNWLLSQQQADG	10.3	13.1	6.4	4.8

SFQDPC(cam)PVLDR				
LQGLEQEAENK	13.3	6.3	5.5	3.1
LTQLNLDR	11.3	14.2	1.9	2.8
LVSLSAQNLVDC(cam)STEK	9.3	13.1	5.2	7.7
NPC(cam)QDPYILTPENR	7.3	17.7	6.2	3.9
QGFILSGVK	12.4	11.6	4.7	3.2
QIGGDGMMDITDTYK	7.7	15.4	4.8	1.6
SGIAYILHLK	10.8	12.3	3.9	3.5
SLGTIQQC(cam)C(cam)DAIDHC(cam)R	6.6	5.1	3.0	4.8
STVLTIPEIIIK	11.3	10.6	4.9	4.3
TDNDGLGFR	17.0	9.3	4.5	3.0
VVEEQESRVHYTVC(cam)IWRNGK	12.4	4.5	4.6	5.4
YTELPYGR	17.9	12.0	6.0	5.4

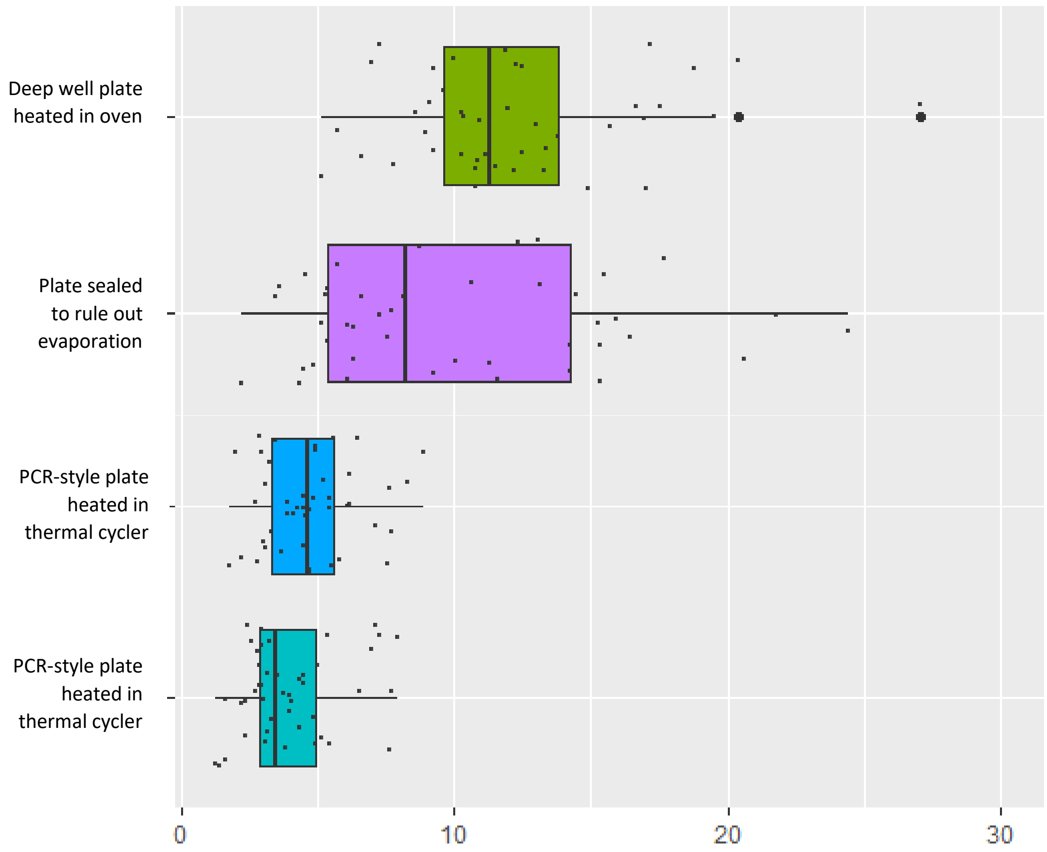

Figure S1. The edge effect and its removal demonstrated in box plots showing the RSDs of the 46 peptides under analysis iteratively improved throughout the course of improved even heating across the plate and normalisation to surrogate.

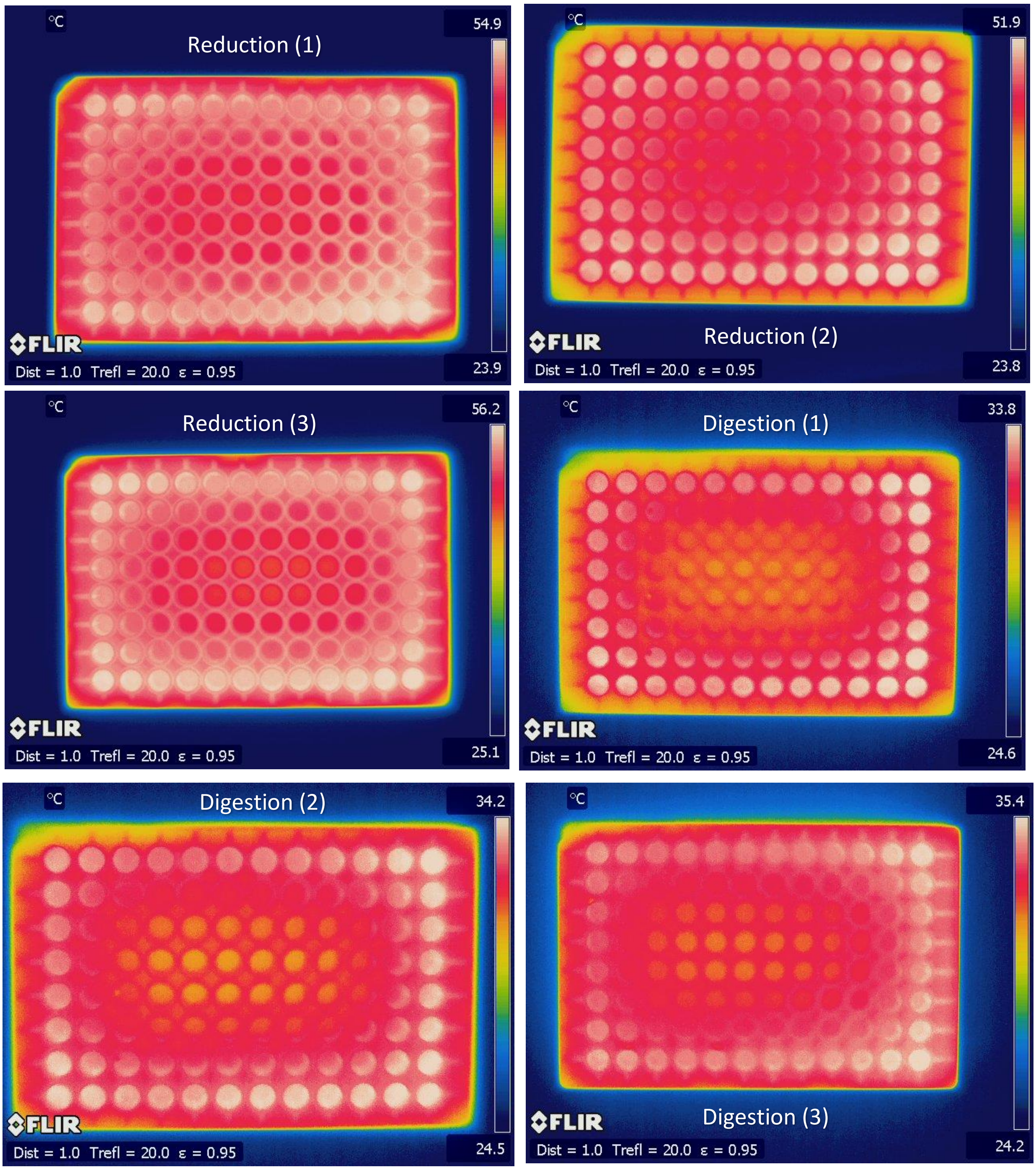

Figure S2. Triplicate IR images showing the temperature distribution across the plates demonstrating temperature gradients (°C) across the multiwell plates as the clear cause of the edge effect in both the reduction steps (first three images) and the tryptic digestion steps (last three images).

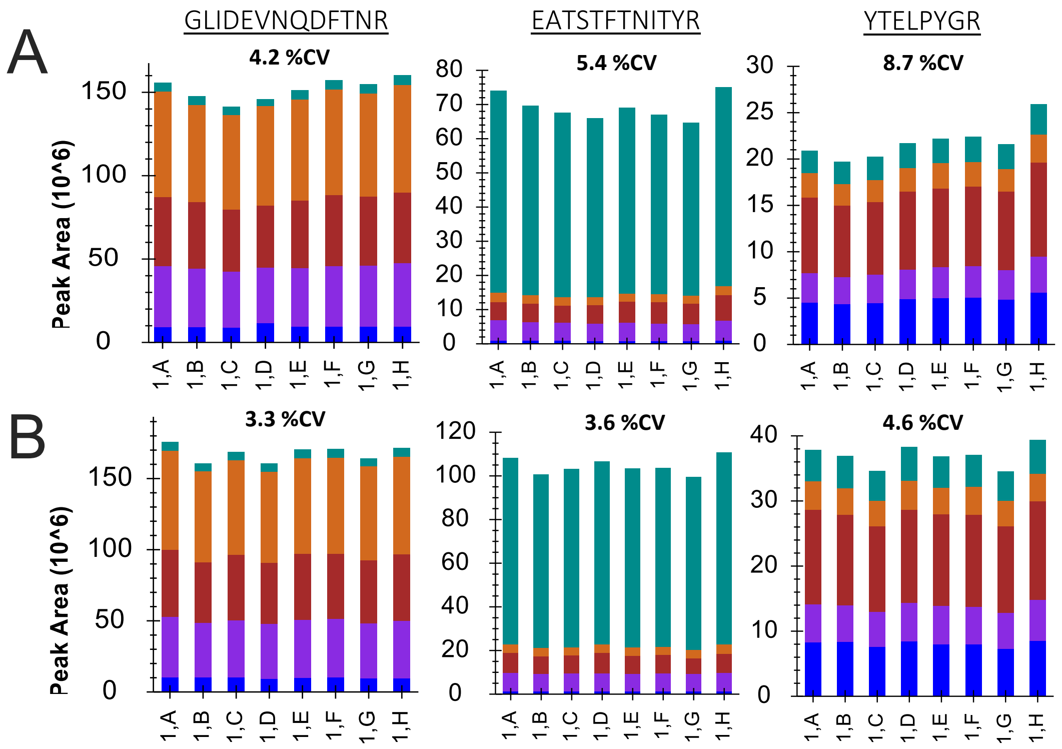

Figure S3. The edge effect demonstrated in peak area bar plots and RSDs of 3 peptides (GLI[…], EAT[…] and YTE[…]) across the first column of the plate i.e. 1,A – 1,H. (**A**) Peak areas demonstrating the presence of the edge effect in experiment 3 (heating with water bath). (**B**) Peak areas for experiment 2, in which the plate was heated with metallic "bath armour" beads in a dry bath.

Table S5. ANOVA and Tukey post-hoc test results comparing the peak areas between each region of the plate: the corner wells, other edge wells (row 1), 2nd from the outside row (row 2), row 3, and centre wells. Significance codes: 0 '***' 0.001 '**' 0.01 '*' 0.05 '.' 0.1 'N/A ' 1.

Comparison	p-value (adjusted)	Significance
**ANOVA All Areas ~ Region**	0.202	N/A
**Corner – Centre**	0.838	N/A
**Row 1 – Centre**	0.774	N/A
**Row 2 - Centre**	0.142	N/A
**Row3 - Centre**	0.581	N/A
**Row 1 – Corner**	0.995	N/A
**Row 2 – Corner**	0.996	N/A
**Row 3 – Corner**	1.000	N/A
**Row 2 – Row 1**	0.503	N/A
**Row 3 – Row 1**	0.988	N/A
**Row 3 – Row 2**	0.873	N/A

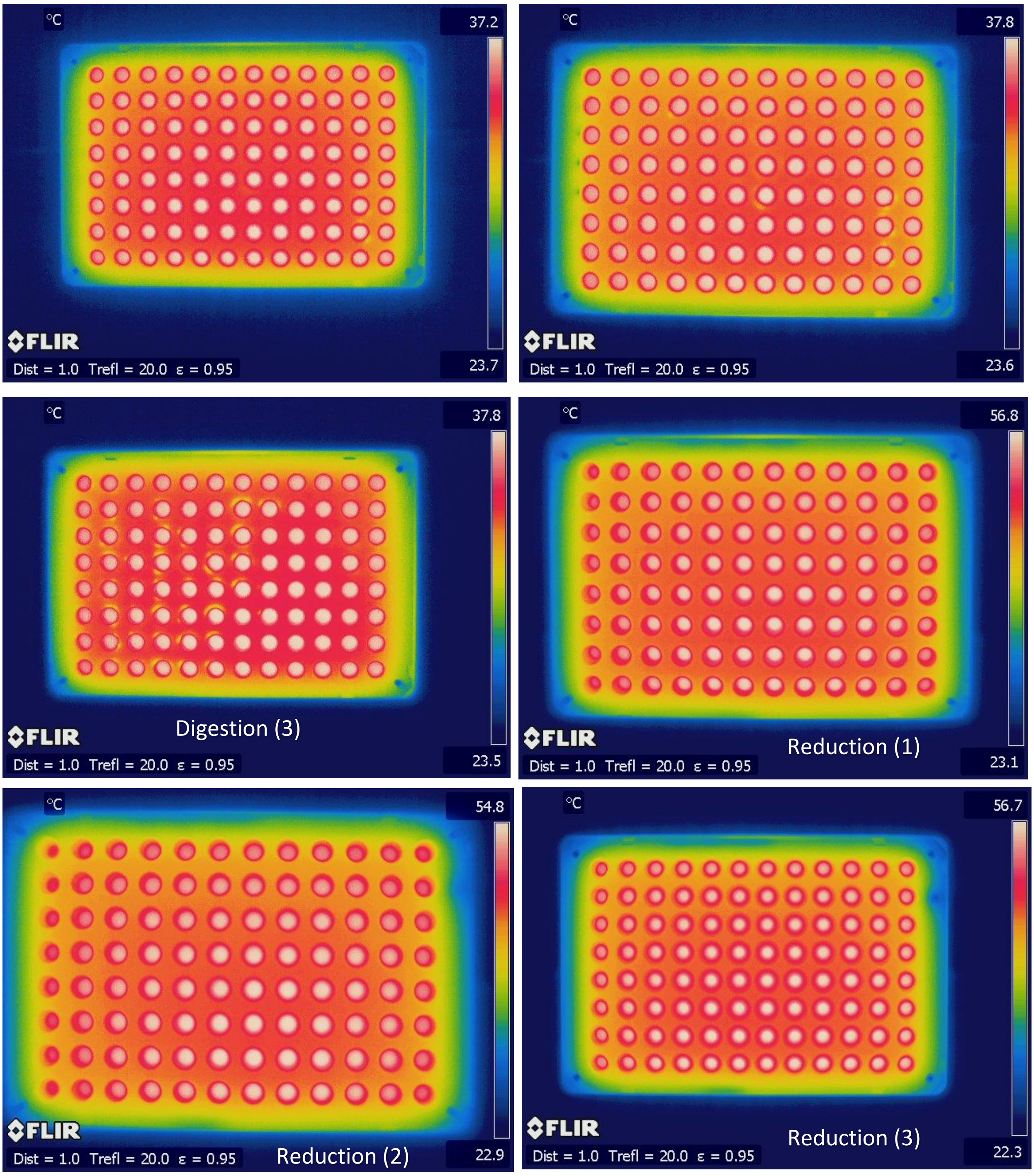

Figure S4. Triplicate IR images showing the temperature distribution across the plates demonstrating even heating across the multiwell plates and thus the amelioration of the edge effect using a PCR-style heating set-up in both the reduction steps (first three images) and the tryptic digestion steps (last three images).
